# Small-Molecule-Based Nanoassemblies as Inducible Nanoprobes for Monitoring Dynamic Molecular Interactions Inside Live Cells**

**DOI:** 10.1002/anie.201101467

**Published:** 2011-07-27

**Authors:** Sangkyu Lee, Kyoung Hu Lee, Jae-Seok Ha, Seung-Goo Lee, Tae K Kim

**Affiliations:** Reons Innovative Medicines InstituteAnyang 431-755 (Korea); Department of Biological Sciences, Korea Advanced Institute of Science and TechnologyDaejeon 305-701 (Korea); Korea Research Institute of Bioscience and BiotechnologyDaejeon 305-806 (Korea)

**Keywords:** fluorescence, molecular interactions, nanoassemblies, nanoparticles, proteins

Monitoring dynamic protein–protein interactions is a fundamental step to elucidate a variety of signaling processes inside cells. Identifying the target proteins of bioactive small molecules is critical to understanding their intracellular action mechanisms. Although several technologies to probe these protein–protein and small-molecule–protein interactions were developed,[1] they suffer from diverse intrinsic problems including high background noise, false positive/negative modes, limited sensitivity and dynamic range, and indirect or delayed readouts. In addition, the use of an artificial milieu, such as in vitro binding conditions or non-mammalian cells, has often led to erroneous experimental outputs.[2] Recently, various types of imaging-based nanotechnologies have emerged to address these limitations.[3] Despite their enormous potential as nanosensors, application of artificially synthesized nanoparticles to probing molecular interactions inside living mammalian cells is somewhat limited by several factors including the prerequisite of efficient and nondisruptive introduction of nanoparticles throughout the cells.[3c, 4]

**Scheme 1:**
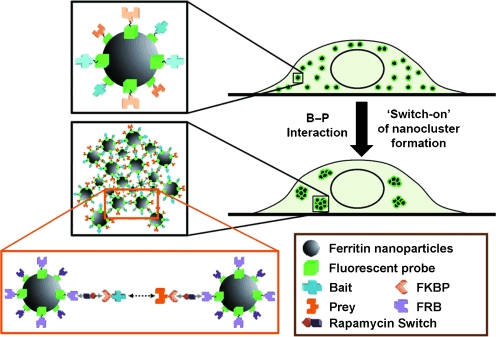
InCell SMART-i. Genetically encoded nanoparticles (NPs) derived from ferritin (FT) are labeled with fluorescent probes (F) for facile detection with fluorescent microscopy. FT-NPs directly or indirectly (as represented in Figure 4 c, for instance) display specific bait (B) or prey (P) molecules inside living cells. Specific B–P interactions induce the interconnected assembly of FP-NPs into nanoclusters which are detected as discrete punctated dots of fluorescence. Using a small-molecule switch, such as rapamycin as an inducer of FKBP-FRB heterodimerization, nanocluster formation can be specifically controlled as shown in Figure 4 c.

Herein, we present a novel phenotypic readout called InCell SMART-i (intracellular supramolecular assembly readout trap for interactions) to directly visualize dynamic molecular interactions within living mammalian cells. Instead of intracellular delivery of artificially synthesized nanoparticles, we employed a strategy to induce the formation of biological nanoparticles by genetic instructions inside living cells. Many organisms have been known to assemble intracellular nanoparticles such as ferritin complexes. It was reported that highly uniform nanoparticles can be assembled by ferritin polymerization under in vitro conditions, and its polymeric state was still retained when expressed in mammalian cells and in vivo.[5] In InCell SMART-i (Scheme 1), genetically engineered ferritin (FT) is used to form nanoparticles (NPs) displaying specific bait (B) or prey (P) molecules inside cells. Inside living cells, the bait molecules on FT-derived nanoparticles (FT-NPs) interact with their target prey molecules. These specific molecular interactions induce the interconnected assembly of FT-NPs into nanoclusters.[6] Consistent with these specific molecular interactions, bait and prey molecules on FT-NPs are co-translocated from the cytoplasm to the newly assembled nanoclusters. Fusion of a fluorescent probe (F) to the bait or prey molecules enables the facile detection of these phenotypic alterations by fluorescent microscopy. These nanocluster formation processes may be cooperative because of the multivalent properties of FT-NPs.[5] In this setting, physical interactions between the bait and prey molecules can be effectively visualized in individual living cells.

**Figure 1:**
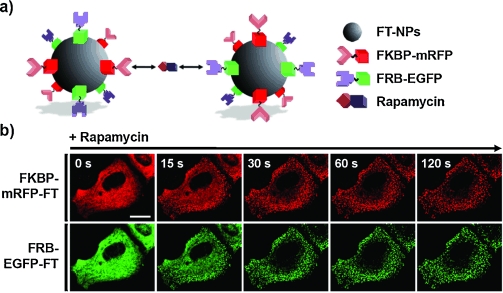
InCell SMART-i for visualizing small-molecule–protein interactions inside living cells. a) InCell SMART-i detecting interactions of rapamycin with FKBP and FRB using ferritin-derived nanoparticles (FT-NPs). b) Time-lapse images of nanocluster formation. Scale bar=20 μm.

To verify this idea, we first examined whether InCell SMART-i could detect specific molecular interactions inside living cells by exploiting rapamycin, a cell-permeable immunosuppressant drug interacting with the FK506 binding protein (FKBP) and with the FKBP-rapamycin binding domain (FRB) of mTOR (Figure 1).[7] Based on this FKBP-FRB heterodimerization, rapamycin has been utilized to rapidly control specific molecular interactions inside cells.[8] To visualize the formation of nanoclusters within cells, monomeric red (mRFP) and enhanced green (EGFP) fluorescent proteins were fused to the FKBP and FRB, respectively (Figure 1 a). HeLa cells were co-transfected with expression plasmids for FKBP-mRFP and FRB-EGFP fused to the N terminus of ferritin (FT). Before induction, nanocluster formation was barely detectable and FT-fusion proteins were mostly dispersed throughout the cytoplasm (Figure 1 b). In contrast, addition of rapamycin induced the heterodimerization of FKBP and FRB displayed on FT-NPs, rapidly driving the assembly of discrete punctated dots of fluorescence within seconds inside cells (Figure 1 b; see also Movie S1 in the Supporting Information). Consistent with FKBP-FRB interactions through rapamycin, we observed the rapamycin-induced co-translocation of FKBP-mRFP-FT and FRB-EGFP-FT from the dispersed cytoplasm to these newly detected punctated fluorescent dots of nanoclusters. These imaging data indicate that interactions of rapamycin with FKBP and FRB induced the assembly of nanoclusters in concomitant with their co-localization onto the nanoclusters in InCell SMART-i.

**Figure 2:**
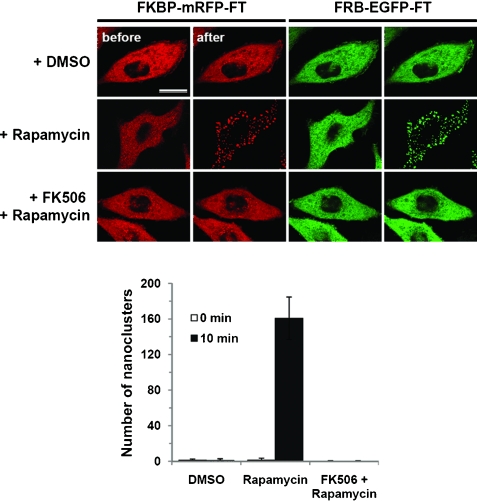
Specific inhibition of nanocluster formation by a competitive inhibitor. HeLa cells were co-transfected with the expression plasmids as indicated. Rapamycin (250 nm) with and without FK506 (25 μm). The images were captured before (0 min) and after (10 min) treatment with rapamycin/FK506. Scale bar=20 μm.

The specificity of nanocluster formation by interactions of rapamycin with FKBP and FRB was further assessed by a series of following experiments. First, nanoclusters were assembled by rapamycin in the presence of both FKBP-mRFP-FT and FRB-EGFP-FT, but neither in the presence of FKBP-mRFP-FT nor in the presence of FRB-EGFP-FT alone (Figure S1 in the Supporting Information). Second, other fluorescent proteins including the enhanced yellow (EYFP) and cyan (ECFP) fluorescent proteins also showed rapamycin-dependent nanocluster formation through heterodimerization of FKBP and FRB on FT-NPs inside cells (Figure S2 in the Supporting Information). Third, FK506, which binds to FKBP but not to FRB, blocked this nanocluster formation as a specific competitor for rapamycin interactions with FKBP and FRB (Figure 2).[7] All of these results support the specificity of the InCell SMART-i technology for molecular interactions inside living cells. Furthermore, the nanocluster formation did not elicit any detectable toxic effects on the cell viability even after long-term (48 h) incubation (Figure S3 in the Supporting Information).

**Figure 3:**
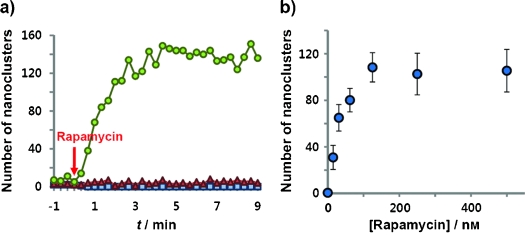
Quantitative analysis of nanocluster formation. a) Temporal analysis of nanocluster formation by FKBP–rapamycin–FRB interactions (rectangles, FKBP; triangles, FRB; circles, FKBP+FRB). b) Dose-dependent nanocluster formation inside living cells.

We next examined the quantitative dynamics of nanocluster formation inside living cells expressing FKBP-mRFP-FT and FRB-EGFP-FT. Nanocluster formation was quantitatively analyzed by the number of assembled nanoclusters with sizes over 0.2 μm^2^ and circularities between 0.5–1.0. Nanocluster formation was initiated as a rapid response to rapamycin induction on a timescale of seconds and became almost saturated within 3–5 min (Figure 3 a; see also Movie S1 in the Supporting Information). This rapamycin-dependent nanocluster formation is specific because it was not observed in the absence of FKBP or FRB (Figure 3 a). The assembly of nanoclusters was augmented with increasing amounts of rapamycin until the saturation point around 100 nm (Figure 3 b). Interestingly, nanocluster formation was dynamically visualized at the subcellular level throughout the cytoplasm on a timescale of seconds (Figure S4 and Movie S1 in the Supporting Information). Notably, high levels of induction in nanocluster formation with high signal-to-noise ratios and distinct phenotypic alterations may facilitate the quantitative analysis of dynamic molecular interactions within live cells (Figure 3; see also Figures 1 and 2). These results suggest that InCell SMART-i can be used for monitoring dynamics of specific interactions of a small molecule with its target protein(s) inside living cells.

**Figure 4:**
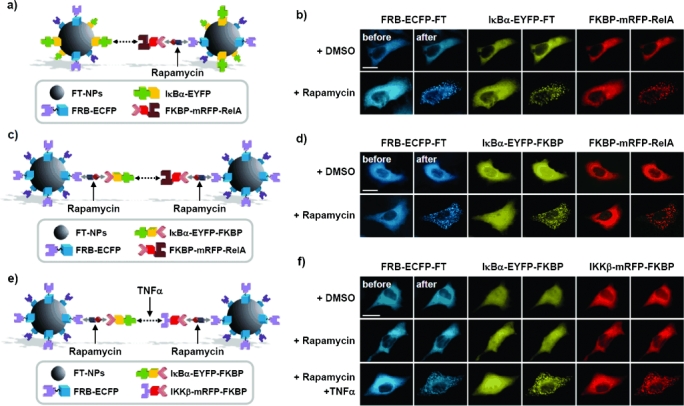
InCell SMART-i for visualizing protein–protein interactions inside living cells. a,c) InCell SMART-i detecting IκBα-RelA interactions using ferritin-derived nanoparticles (FT-NPs). b,d) Specific nanocluster formation by IκBα-RelA interactions. e) InCell SMART-i detecting TNF-α-dependent IκBα-IKKβ interactions using ferritin-derived nanoparticles (FT-NPs). b,d,f) HeLa cells co-transfected with the expression plasmids as indicated were treated with DMSO or rapamycin (500 nm for b,d; 50 nm for f). The images were captured before (0 min) and after (10 min) rapamycin treatment. f) Specific nanocluster formation by IκBα-IKKβ interactions in response to TNF-α (10 ng mL^−1^). Scale bars=20 μm.

Furthermore, we evaluated the feasibility of InCell SMART-i for probing protein–protein interactions in the intracellular signaling processes using the NF-κB pathway.[9] Members of the NF-κB family (e.g., RelA/p65) are sequestered within the cytoplasm by the IκB family member (e.g., IκBα). Various stimuli, including TNF-α, induce the phosphorylation of IκBα by IKKβ. To detect RelA-IκBα interactions in cells, we tested two related formats for monitoring nanocluster formation that are inducible in a rapamycin-dependent manner, extended from our results in Figure 1. As depicted in Figure 4 a,c, IκBα or/and RelA were directly or indirectly displayed on FT-NPs using rapamycin-dependent heterodimerization of FKBP and FRB in HeLa cells. Consistent with RelA-IκBα interactions, rapamycin elicited a bridging function to induce the interconnected assembly of nanoclusters in live cells (Figure 4 b,d). Consistently, addition of rapamycin rapidly induced co-translocation of RelA/IκBα-fusion proteins from the cytoplasm to the newly assembled nanoclusters. These results suggest that rapamycin may be useful as a small-molecule switch to inducibly visualize nanocluster formation for probing intracellular molecular interactions in InCell SMART-i. This inducible system with an immediate readout on a timescale of seconds may allow reliable detection for molecular interactions by eliminating nonspecific interactions or aggregates within living cells.

We also examined whether InCell SMART-i can be used to probe signal-dependent interactions of a kinase with its substrate, which are mostly weak and transient, by monitoring TNF-α-induced interactions of IKKβ with IκBα. Both IKKβ and IκBα were indirectly displayed on FT-NPs using rapamycin-dependent heterodimerization of FKBP and FRB in HeLa cells (Figure 4 e). Prior to TNF-α stimulation, formation of nanoclusters was barely detectable. Treatment with rapamycin along with TNF-α inducibly visualized nanocluster formation (Figure 4 f), reflecting signal-dependent interactions of IKKβ kinase with its substrate IκBα inside live cells. Thus, this sensitive system allowed detection of specific and transient protein–protein interactions in response to external stimuli, further emphasizing the specificity of the InCell SMART-i technology inside live cells.

Next, to test whether InCell SMART-i can be used to probe molecular interactions in other intracellular compartments such as nucleus or plasma membranes, we targeted FT-NPs to the nucleus or plasma membranes by using the nuclear localization signal (NLS) or pleckstrin homology (PH) domain of Akt which is known to bind phosphatidylinositol (3,4,5)-triphosphate (PIP3) in the plasma membrane.[10] We inserted NLS into FKBP-ECFP-FT and FRB-mRFP-FT, and the PH domain into FKBP-mRFP-FT and FRB-EGFP-FT (Figures S5a and S6 in the Supporting Information). Consistent with cytosolic interactions as demonstrated in Figure 1, addition of rapamycin rapidly induced the formation of nanoclusters in the nucleus or plasma membranes in concomitant with co-translocation of FKBP and FRB from the nucleus or plasma membranes to the newly assembled nanoclusters.

We further examined the feasibility of InCell SMART-i in visualizing molecular interactions inside the nucleus by exploiting protein–protein interactions of p53 with MDM2 in the nucleus (Figure S5b in the Supporting Information).[11] Following the scheme dipicted in Figure 4 c, p53 and MDM2 were indirectly displayed on nuclear-localized FT-NPs using rapamycin-dependent heterodimerization of FKBP and FRB. Upon treatment with rapamycin, nanoclusters were rapidly assembled by specific interactions of p53 with MDM2 inside the nucleus, demonstrating the spatial resolution of InCell SMART-i for intracellular molecular interactions (see also Figure S4 and Movie S1 in the Supporting Information).

Detection of the interactions of a bioactive small molecule with its target protein(s) is essential for understanding its intracellular action mechanisms as well as its therapeutic and/or deleterious effects.[1, 12] We determined whether InCell SMART-i can detect the intracellular target of a small drug molecule inside living cells by exploiting methotrexate (MTX), an anti-cancer drug interacting with its therapeutic target protein of dihydrofolate reductase (DHFR, Figure S7 in the Supporting Information).[13] HeLa cells were co-transfected with expression plasmids for FKBP, FRB, and DHFR fused to mRFP-FT, displaying FKBP, FRB, and DHFR on FT-NPs within cells. As demonstrated in Figure 1, rapamycin rapidly induced formation of nanoclusters. Under these conditions, we observed specific recruitment of fluorescence-labeled MTX (BODIPY FL-MTX) onto assembled nanoclusters (Figure S7 in the Supporting Information). In contrast, this recruitment of BODIPY FL-MTX was not observed in nanoclusters displaying no DHFR. These results suggest that InCell SMART-i will be useful for identifying targets of small-molecule modulators from phenotypic screening for development of molecular probes by biochemical approaches.[1, 12]

In summary, we have developed a novel and effective readout for specific molecular interactions inside living cells by monitoring the induced assembly of nanoclusters. InCell SMART-i may offer several advantages compared to traditional technologies. First, it probes biomolecular interactions in a physiologically and pharmacologically relevant context, and thereby greatly diminishes misleading outcomes produced by artificial experimental settings.[1–2] Second, InCell SMART-i directly translates physical interactions into clear readout signals, unlike indirect readout methods that rely on complex gene expression or biological phenotypic profiles.[1] Third, InCell SMART-i is amenable to dynamic, single-cell analysis of interactions. Fourth, InCell SMART-i is simpler and easier to perform relative to the artificially synthesized nanoparticles, which face practical issues such as facile intracellular delivery,[3c, 4] because of the use of biologically self-assembled ferritin complexes inside cells. Fifth, rapid detection systems are feasible because high signal-to-noise ratios are observed and inducible by small molecules. Therefore, InCell SMART-i may be exploited in systematic screening of diverse molecular interactions inside living cells, as it efficiently eliminates intrinsic false positive/negative modes or error-prone deviations. Furthermore, InCell SMART-i with distinct phenotypic readouts may complement some limitations of readouts based on fluorescence intensities for probing molecular interactions such as fluorescence resonance energy transfer (FRET) or bimolecular fluorescence complementation (BiFC), the shortcomings of which are limited sensitivity and dynamic range or long maturation times of fluorophores, respectively.[14] With these great advantages, our current efforts include genome-wide interaction screens for small-molecule target identification and the systematic analysis of signal transduction networks.

In conclusion, InCell SMART-i will be useful for probing dynamic molecular interactions inside living cells with its technological versatility. Detailed molecular mechanisms underlying supramolecular assembly of nanoclusters inside cells remain to be determined, although similar types of nanoassemblies were also observed in vitro.[6] In addition, the use of super-resolution microscopy and other technologies for small fluorescent tags and small-molecule dimerizers might further facilitate high-resolution imaging of dynamic molecular interactions and effective use of InCell SMART-i.[3a, c]
