# Supplementary material for: Small-Molecule-Based Nanoassemblies as Inducible Nanoprobes for Monitoring Dynamic Molecular Interactions Inside Live Cells
Source: Angew Chem Int Ed Engl. 2011 Jul 27;50(37):8709–13. doi: 10.1002/anie.201101467 (PMC3229982; doi:10.1002/anie.201101467)
Supplement: Supplementary file 1 [file anie0050-8709-SD1.pdf]

Angewandte Chemie
Eine Zeitschrift der Gesellschaft Deutscher Chemiker

Supporting Information

© Wiley-VCH 2011

69451 Weinheim, Germany

Small-Molecule-Based Nanoassemblies as Inducible Nanoprobes for Monitoring Dynamic Molecular Interactions Inside Live Cells**

*Sangkyu Lee, Kyoung Hu Lee, Jae-Seok Ha, Seung-Goo Lee, and Tae K. Kim**

anie_201101467_sm_miscellaneous_information.pdf

Contents

Experimental Section --- **S2**

References -- **S4**

Figure S1. Nanocluster formation by interactions of rapamycin with FKBP and FRB inside living cells. -- **S5**

Figure S2. Nanocluster formation by ferritin (FT)-derived nanoparticles labeled with various fluorescent proteins inside living cells. ----------------------------------- **S6**

Figure S3. Effect of nanocluster formation on cell viability. ------------------------- **S7**

Figure S4. Monitoring dynamics of molecular interactions inside living cells in InCell SMART-i. --- **S8**

Figure S5. InCell SMART-i for visualizing molecular interactions inside nucleus. **S9**

Figure S6. InCell SMART-i for visualizing molecular interactions in plasma membrane. -- **S10**

Figure S7. InCell SMART-i for visualizing specific interactions of methotrexate (MTX) with dihydrofolate reductase (DHFR). -- **S11**

Movie S1. Specific nanocluster formation visualizing small molecule (rapamycin) and protein (FKBP and FRB) interactions inside living cells.

Experimental Section

DNA constructions

cDNA encoding human ferritin light subunit (FT; GeneBank Acc. No. BC016346) was purchased from Open BioSystems (Huntsville, AL, USA) and subcloned into pcDNA3.1(+)/Zeo (Invitrogen) to generate various FT expression plasmids. EGFP excised by *BamHI* and *BsrGI* from EGFP-N1 (Clontech) was fused to the N-terminus of FT for EGFP-FT plasmid. EGFP in EGFP-FT was replaced with mRFP (excised from ref. [1]), EYFP (excised from EYFP-N1 (Clontech)), and ECFP (excised from ECFP-N1 (Clontech)) for mRFP-FT, EYFP-FT, and ECFP-FT plasmids, respectively. cDNAs of FKBP12 (FKBP) and FRB flanked by *EcoRI* and *BamHI* were fused to the N-termini of mRFP-FT, EYFP-FT, EGFP-FT, and ECFP-FT for FKBP-mRFP-FT, FRB-EYFP-FT, FRB-EGFP-FT, FKBP-ECFP-FT, and FRB-ECFP-FT plasmids. cDNA of IκBα (excised from ref. [2]) flanked by *HindIII* and *BamHI* was fused to the N-terminus of EYFP-FT for IκBα-EYFP-FT plasmid. FKBP-mRFP was fused to the N-terminus of EYFP-RelA (ref. [3]) after excision of EYFP by *NheI* and *BsrGI* for FKBP-mRFP-RelA plasmid. IκBα-EYFP-FKBP plasmid was generated by replacing FT with FKBP in IκBα-EYFP-FT. EYFP in EYFP-FKBP vector was replaced with mRFP to make mRFP-FKBP expression vector. Plasmids for IKKβ-mRFP-FKBP and MDM2-mRFP-FKBP were generated by conjugating IKKβ and MDM2 at the N-terminus of mRFP-FKBP, respectively. With EYFP-C1 (Clonetech), FKBP flanked by *NheI* and *AgeI* and p53 (R248W) flanked by *BglII* and *HindIII* were fused at the N-terminus and C-terminus of EYFP, respectively, to generate FKBP-EYFP-p53 expression plasmid. ECFP-NLS-C1 vector encoding ECFP tagged with nuclear localization signal (NLS) was generated by inserting multicloning site into the C-terminus of ECFP-Nuc vector (Clontech). By inserting FT into the C-terminus of ECFP-NLS with *EcoRI* and *BamHI*, ECFP-NLS-FT expression plasmid was generated. ECFP in ECFP-NLS-FT was replaced with mRFP to make mRFP-NLS-FT expression plasmid. FKBP-ECFP-NLS-FT and FRB-mRFP-NLS-FT were subcloned by conjugating FKBP and FRB which are flanked by *NheI* and *AgeI* at the N-terminus of ECFP-NLS-FT and mRFP-NLS-FT, respectively. PH domain of Akt flanked by *NheI* and *HindIII* was inserted into N-termini of FKBP-mRFP-FT and FRB-EGFP-FT for PH-FKBP-mRFP-FT and PH-FRB-EGFP-FT, respectively. cDNA of dihydrofolate reductase (DHFR, from *Escherichia Coli*) flanked by *NheI* and *XhoI* was fused to the N-terminus of mRFP-FT for DHFR-mRFP-FT expression vector.

InCell SMART-i assay

HeLa cells were obtained from American Type Culture Collection (ATCC) and maintained in DMEM (Invitrogen) supplemented with 10 % fetal bovine serum (Invitrogen). Transfection was performed using a Microporator^{TM} (MP-100; Digital Bio Technology) according to the manufacturer's instructions. In the optimized condition, 2 pulses of electric shock at 980 V were transferred to HeLa cells for 35 milliseconds. At 24 hours after transfection, cells were washed with OPTI-MEM (Invitrogen) and treated with selected concentrations of rapamycin (Calbiochem), FK506 (Sigma), BODIPY FL-MTX (Molecular Probes) or TNF-α (Sigma) as described in the manuscript. For competition experiments, transfected cells were pre-incubated with FK506 for 10 minutes before treatment with rapamycin. To analyze interactions of MTX with DHFR, transfected cells were pre-treated with BODIPY FL-MTX for 1 hour before treatment with rapamycin. The rapamycin, FK506, and BODIPY FL-MTX were dissolved in DMSO as 2 mM stock solution and diluted in OPTI-MEM before their use. TNF-α was dissolved in distilled water to a concentration of 10 μg /ml for stock solution and diluted in OPTI-MEM before treatment.

Fluorescent and confocal imaging

Fluorescent microscopy was performed on the Olympus IX51 with a LUC Plan FL N objective lens (40x/0.6NA). Confocal microscopy was performed on the Nikon A1R with a CFI Plan Apochromat VC objective lens (60x/1.40NA) along with digital zooming of NIS-element software (Nikon) according to the manufacturer's instructions.

Image processing and analysis

Images were stored as JPG and ND2 (or JPEG2000) file formats after capturing by fluorescent and confocal microscopy, respectively. Cropping of images was performed with Adobe Photoshop CS3 or MetaMorph offline version 7.6.0.0 (MDS Analytical Technologies). ND2 files were converted into ICS format and "Make Movie" tool in the MetaMorph software was used to make video files. Nanoclusters were defined as discrete punctated dots of fluorescence with criteria of fluorescent intensity (2500-4095), size (>0.2 μm^2), and circularity (0.5-1.0). Number of nanoclusters per cell was measured with "Object Count" tool in Nikon imaging software (NIS-element AR 64-bit version 3.00, Laboratory Imaging). To quantitatively analyze the propagation of nanocluster formation, "Intensity Surface Plot" tool in the NIS-element software was used. Colocalization of MTX and nanoclusters was quantified with Pearson's correlation coefficient by using "Colocalization" tool in Nikon imaging software.

Statistical Analysis

Statistical analysis was performed using an unpaired *t*-test.

References

[1] R. E. Campbell, O. Tour, A. E. Palmer, P. A. Steinbach, G. S. Baird, D. A. Zacharias, R. Y. Tsien, *Proc Natl Acad Sci U S A* **2002**, *99*, 7877.

[2] T. Seo, J. Park, C. Lim, J. Choe, *Oncogene* **2004**, *23*, 6146.

[3] J. A. Schmid, A. Birbach, R. Hofer-Warbinek, M. Pengg, U. Burner, P. G. Furtmuller, B. R. Binder, R. de Martin, *J Biol Chem* **2000**, *275*, 17035.

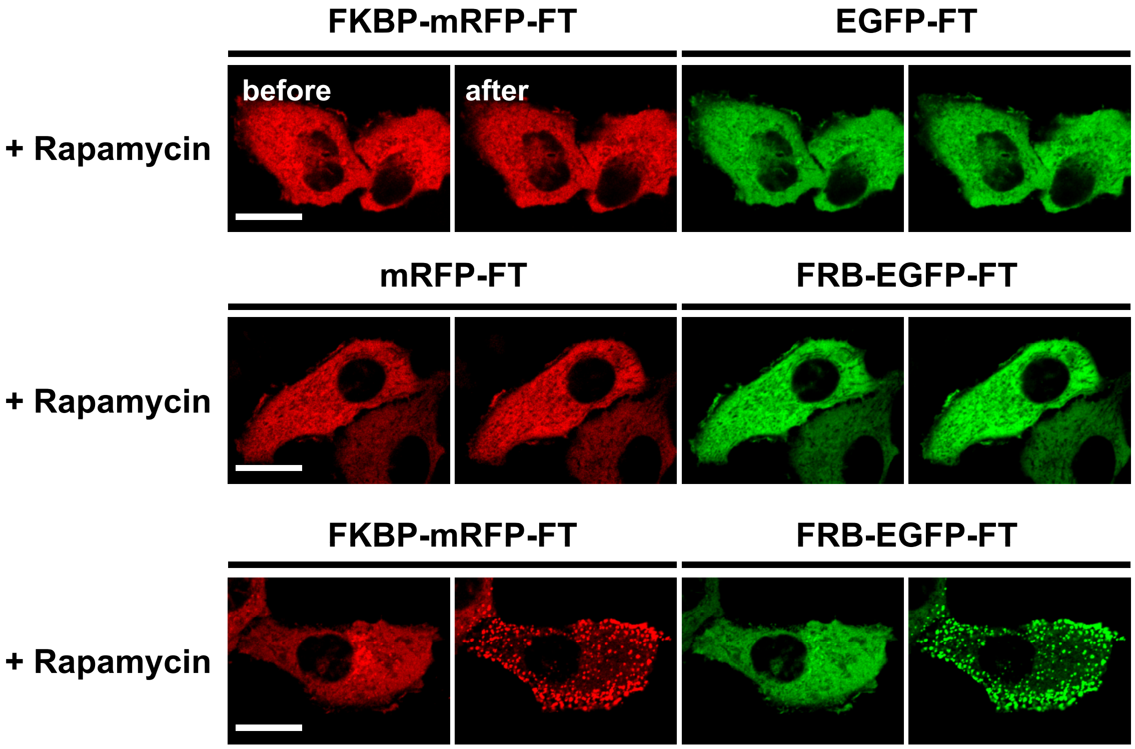

Figure S1. Nanocluster formation by interactions of rapamycin with FKBP and FRB inside living cells. HeLa cells were co-transfected with the expression plasmids as indicated and then treated with 500 nM rapamycin. The images were captured before (0 min) and after (10 min) rapamycin treatment. Scale bar = 20 μm.

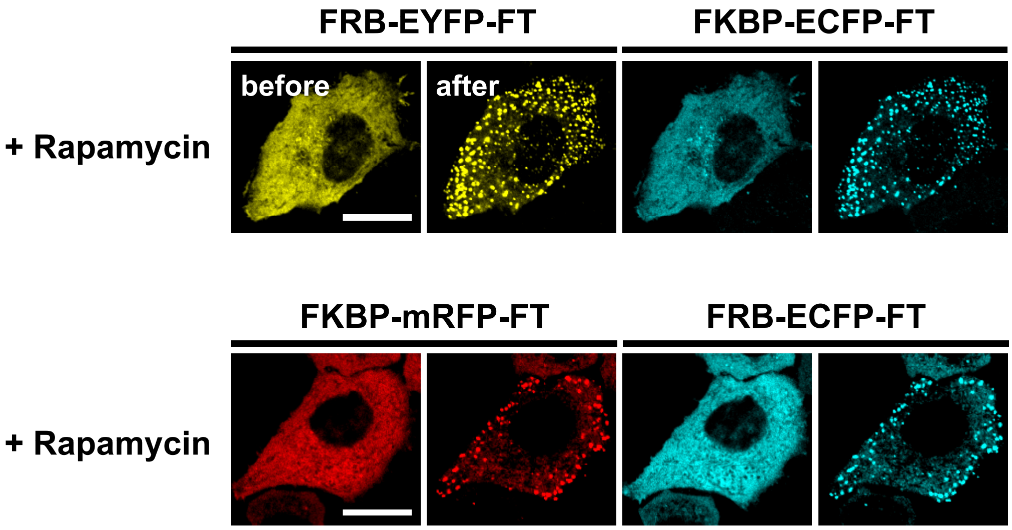

Figure S2. Nanocluster formation by ferritin (FT)-derived nanoparticles labeled with various fluorescent proteins inside living cells. HeLa cells were co-transfected with the expression plasmids as indicated and then treated with 500 nM rapamycin. The images were captured before (0 min) and after (10 min) rapamycin treatment. Scale bar = 20 μm.

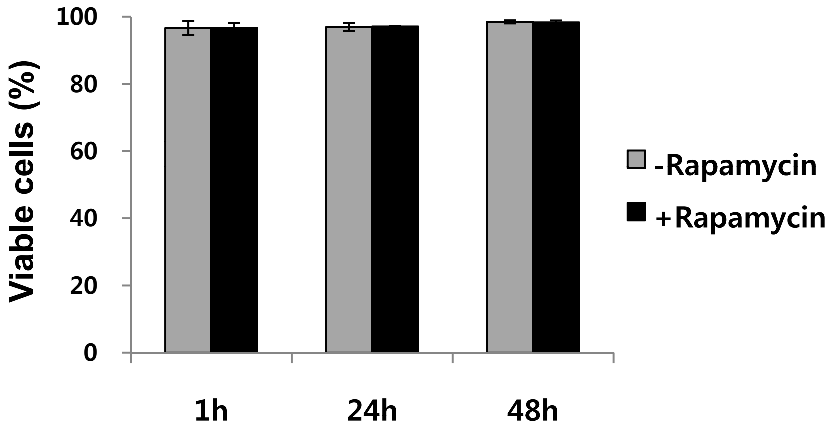

Figure S3. Effect of nanocluster formation on cell viability. HeLa cells co-transfected with the expression plasmids for FKBP-mRFP-FT and FRB-EGFP-FT were treated with 500 nM rapamycin for three different incubation periods (1, 24, 48 h). Treated cells were harvested and stained with trypan blue to measure the percentages of viable cells.

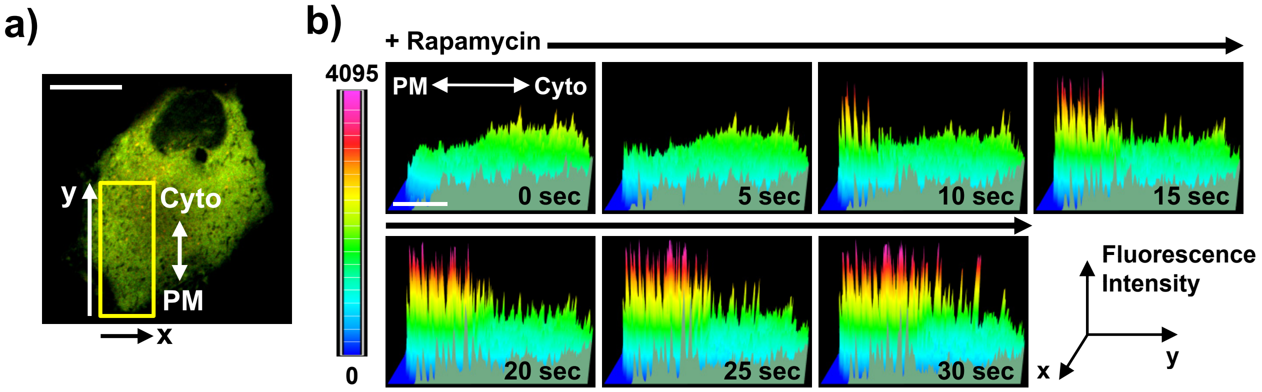

Figure S4. Monitoring dynamics of molecular interactions inside living cells in InCell SMART-i. a) Merged image for a cell co-expressing FKBP-mRFP-FT and FRB-EGFP-FT. Scale bar = 20 µm. b) Intensity surface plotting of nanocluster formations propagated from plasma membrane (PM) into cytoplasm (Cyto). Fluorescence intensity was shown in pseudocolor. Analyzed region is indicated by the yellow box in panel a.

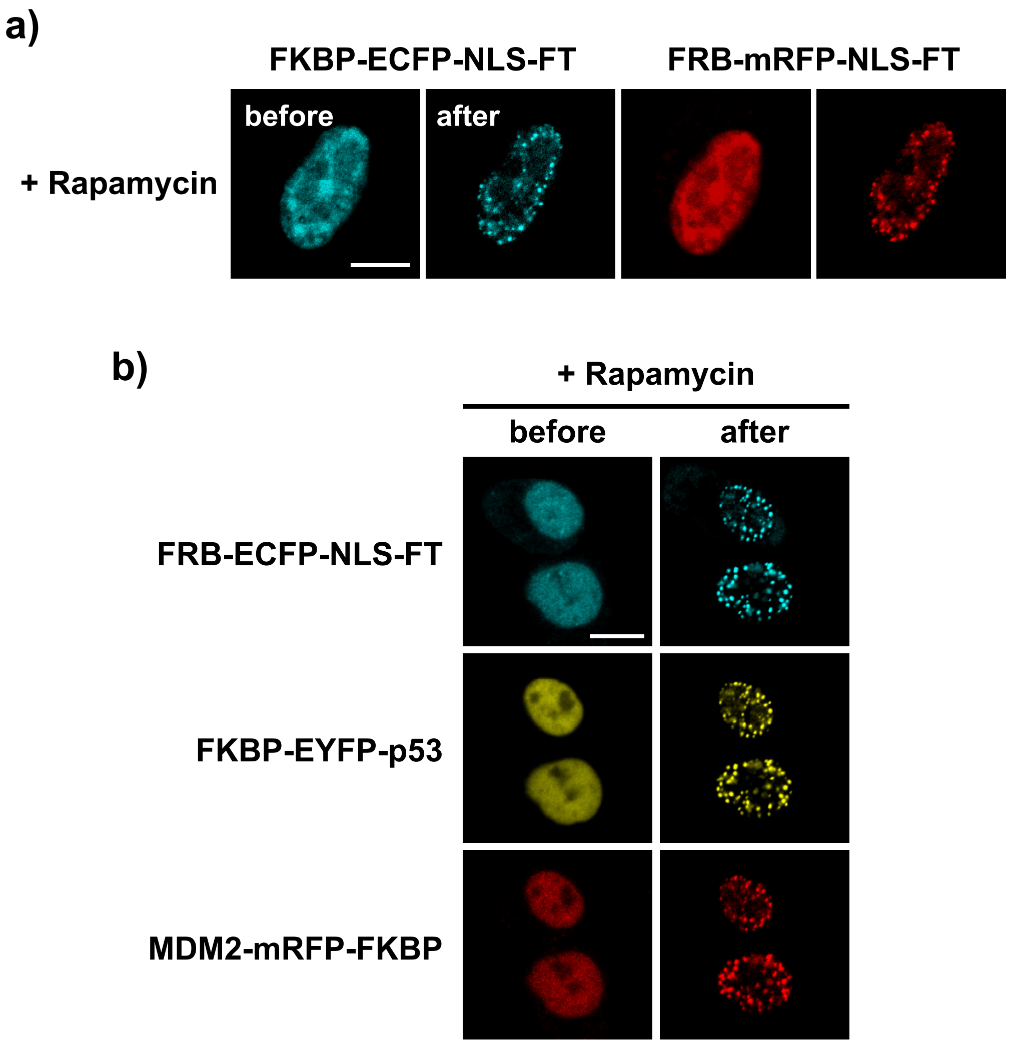

Figure S5. InCell SMART-i for visualizing molecular interactions inside nucleus. HeLa cells co-transfected with the expression plasmids as indicated were treated with 500 nM rapamycin. The images were captured before (0 min) and after (10 min) rapamycin treatment. a) Nanocluster formation by interactions of rapamycin with FKBP and FRB. b) Nanocluster formation by interactions of p53 with MDM2. Experimental scheme was same as shown in Figure 4c. Scale bar = 10 μm.

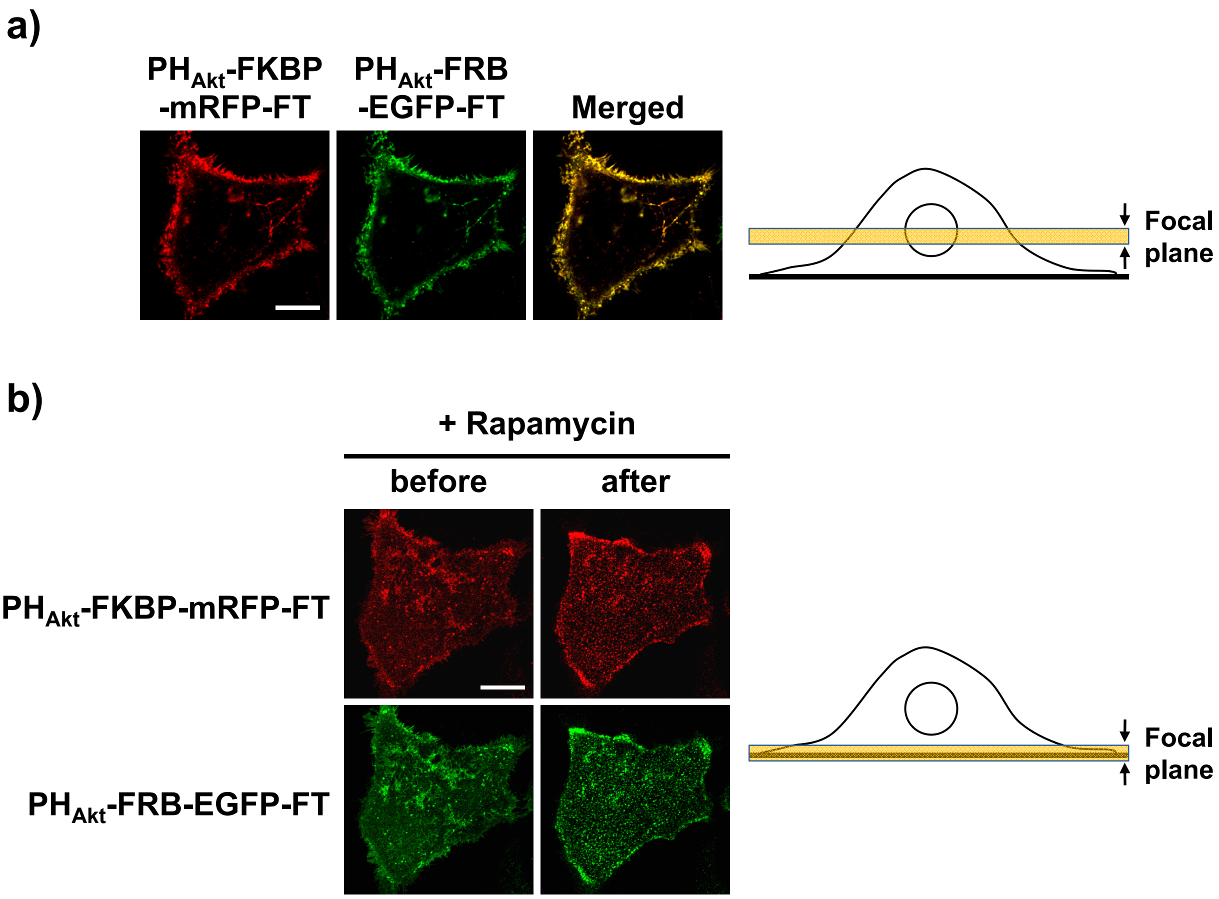

Figure S6. InCell SMART-i for visualizing molecular interactions in plasma membrane. HeLa cells co-transfected with the expression plasmids as indicated were treated with 500 nM rapamycin. The images were captured before (0 min) and after (10 min) rapamycin treatment. a) Fluorescent images of HeLa cell were captured after adjusting the focal plane to the middle of the cell before rapamycin treatment. b) Nanocluster formation by interactions of rapamycin with FKBP and FRB in the plasma membrane. Images were captured after adjusting the focal plane to the basal membrane of the cell. Scale bar = 20 μm.

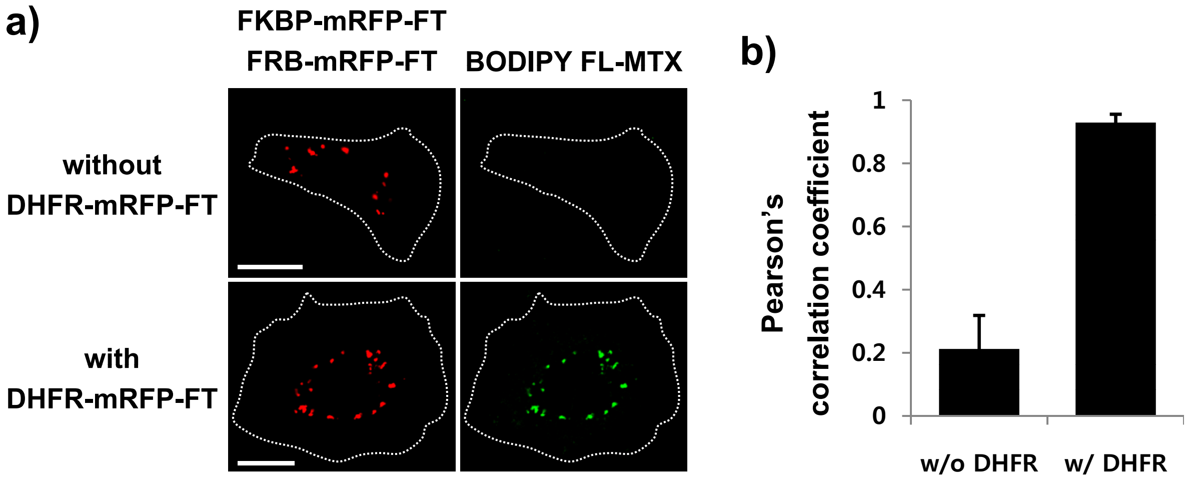

Figure S7. InCell SMART-i for visualizing specific interactions of methotrexate (MTX) with dihydrofolate reductase (DHFR). a) HeLa cells co-transfected with the expression plasmids as indicated were treated with 500 nM rapamycin and 10 μM BODIPY FL-MTX. The images were captured at 2 hrs after chemical treatment. Dashed line indicates cell boundary. Scale bar = 20 μm. b) Quantitative analysis of colocalization of mRFP-labeled nanoclusters with BODIPY FL-labeled MTX.
